# Lithium Carbonate in the Treatment of Graves' Disease with ATD-Induced Hepatic Injury or Leukopenia

**DOI:** 10.1155/2015/694023

**Published:** 2015-10-20

**Authors:** Rendong Zheng, Kemian Liu, Kun Chen, Wen Cao, Lin Cao, Huifeng Zhang, Hongping Sun, Chao Liu

**Affiliations:** Department of Endocrinology and Metabolism, Jiangsu Province Hospital on Integration of Chinese and Western Medicine, Nanjing University of Traditional Chinese Medicine, 100 Shizi Street, Hongshan Road, Nanjing, Jiangsu 210028, China

## Abstract

*Objective*. GD with ATD-induced hepatic injury or leukopenia occurs frequently in clinical practice. The purpose of the present study was to observe the clinical effect of lithium carbonate on hyperthyroidism in patients with GD with hepatic injury or leukopenia. *Methods*. Fifty-one patients with GD with hepatic injury or leukopenia participated in the study. All patients were treated with lithium carbonate, in addition to hepatoprotective drugs or drugs that increase white blood cell count. Thyroid function, liver function, and white blood cells were measured. Clinical outcomes were observed after a 1-year follow-up. *Results*. After treatment for 36 weeks, symptoms of hyperthyroidism and the level of thyroid hormones were improved and liver function, and white blood cells returned to a normal level. Twelve patients (23.5%) obtained clinical remission, 6 patients (11.8%) relapsed after withdrawal, 25 patients (49.0%) received radioiodine therapy, and 8 patients (15.7%) underwent surgical procedures after lithium carbonate treatment. *Conclusion*. Lithium carbonate has effects on the treatment of mild-to-moderate hyperthyroidism caused by GD, and it is particularly suitable for patients with ATD-induced hepatic injury or leukopenia.

## 1. Introduction

After sixty years of development, the treatment of hyperthyroidism remains dependent upon three measures including antithyroid drugs (ATDs) and radioiodine and surgery [1]. Currently, methimazole (MMI) and propylthiouracil (PTU) are the most commonly prescribed ATDs. Side effects of ATD frequently appear in clinical practice and include rash, fever, hepatic injury [2–4], and leukopenia or agranulocytosis [5–8].

Immunoallergic hepatitis is also a common side effect of ATD therapy, affecting 1% of patients treated with ATD [5]. A transient increase in aminotransferase levels is observed in 30% of patients taking PTU [2]. Leukopenia or agranulocytosis is a severe side effect of ATD. One study reported that the use of ATD causes agranulocytosis in 0.1–0.5% of treated patients [5]. Clinical studies have found that agranulocytosis occurred in 0.37% of patients who received PTU and in 0.35% who received MMT [9].

Lithium carbonate has been used in the treatment of mania and in the prophylaxis against recurrent manic-depressive disorders, but it is also used as an adjunct drug for the treatment of hyperthyroidism in clinic [10]. Lithium carbonate inhibits the release of thyroid hormones and inhibits the synthesis of thyroid hormones [11, 12]. It has been reported frequently that patients with Graves' Disease (GD) were treated with lithium carbonate in combination with radioiodine [13, 14]. However, the potential toxicity of lithium limits its application in hyperthyroidism, and its clinical effectiveness has not received much attention.

This is the first report to study the effects of lithium carbonate on patients with GD as well as hepatic injury or leukopenia. In the study, 51 GD patients with hepatic injury or leukopenia were treated with lithium carbonate. The aims of this study were to investigate the clinical effectiveness of lithium carbonate on hyperthyroidism and to evaluate its safety.

## 2. Patients and Methods

### 2.1. Study Patients

Fifty-one GD patients with hepatic injury or leukopenia were recruited from January 2010 to January 2014 in department of endocrinology and metabolism. Informed consent was obtained from all patients prior to the lithium carbonate treatment. The study was approved by the ethics committee of the Jiangsu Province Hospital on Integration of Chinese and Western Medicine, Nanjing University of Traditional Chinese Medicine. The patients included 8 males and 43 females and were 20–58 years of age. According to liver function and leukocyte count, 51 patients with GD were enrolled in this study, including 33 GD patients with hepatic injury (5 males and 28 females, 64.7%) and 18 GD patients with leukopenia (3 males and 15 females, 35.3%) (Table 1). We excluded the patients with thyroid crisis, who were pregnant woman, with psoriasis or a history of psoriasis, with renal insufficiency, with severe heart failure (NYHA Functional Classification is more than Class III), with hepatic injury or leukopenia that have been induced by other reasons and patients who have contraindications of lithium carbonate.

#### 2.1.1. Diagnostic Criteria

*GD*. The signs and symptoms of hypermetabolism due to thyrotoxicosis, a diffuse goiter, increasing free T4 and decreasing TSH levels, an elevated radioiodine uptake, with or without orbitopathy and thyroid autoantibodies (TRAb, TPOAb, and TGAb).

*GD with Hepatic Injury*. The concentration of ALT (aminotransferase) and/or AST (aspartate aminotransferase) exceeded 2 times that of a normal lever and exclusion of viral hepatitis and other liver diseases.

*GD with Leukopenia*. A decrease in the circulating WBC (white blood cell) count to less than 4.0 × 10^9^/L, and agranulocytosis is defined by a reduction in the peripheral neutrophil count to less than 0.5 × 10^9^/L and exclusion of hematological system disease.

All patients were followed up for at least 1 year after stopping treatment; thyroid function (FT3, FT4, and TSH), liver function (total bilirubin, ALT, and AST), and white blood cell count were measured every 4 weeks.

A diagnosis of remission and relapse was based on the following criteria. Patients were considered to be in remission if the FT3 and FT4 level were within the normal range at the last visit. Patients were considered to have relapsed if the FT3 and FT4 level exceeded the upper limit of the normal range and TSH levels were low during the follow-up.

### 2.2. Treatment Method

Fifty-one patients with antithyroid drug-induced hepatic injury or leukopenia were treated with lithium carbonate for 36 weeks. On the other hand, GD patients with hepatic injury were treated with a hepatoprotective drug, and patients with leukopenia were treated with drugs to increase white blood cells, simultaneously (Table 2).

### 2.3. Laboratory Evaluation

Thyroid function, liver function, and white blood cell counts were measured every 4 weeks. Thyroid function test was as follows: FT3 (normal range: 3.50–6.50 pmol/L), FT4 (normal range: 11.50–22.70 pmol/L), TSH (normal range: 0.55–4.78 mIU/L), and TRAb, TGAb, and TPOAb (Siemens Healthcare Diagnostics, New York, USA) were detected by chemiluminescence immunoassay. Liver function test was as follows: alanine aminotransferase (ALT, normal range: <40 U/L), aspartate aminotransferase (AST, normal range: <40 U/L), total bilirubin (TBIL, normal range: 8.50–21.0 *μ*mol/L), (Roche Diagnostics, Mannheim, Germany), and white blood cells (WBC, normal range: 4.0–10.0 × 10^9^/L), and neutrophils (N, normal range: 2.5–7.5 × 10^9^/L) were assessed.

### 2.4. Serum Lithium Concentration

Serum lithium concentration was measured by a colorimetric assay (Roche Diagnostics, Mannheim, Germany).

### 2.5. Side Effects

Side effects were observed during treatment, such as neuropsychiatric disorders, rash, nausea, vomiting, altered kidney function, and abnormality of blood glucose.

### 2.6. Statistical Methods

The data analysis was evaluated using SPSS 16.0; *p* values < 0.05 were considered statistically significant. All values are expressed as the mean ± SD for the quantitative variables and as a percentage for the qualitative variables. The characteristics of the two groups were compared by *t*-test or nonparametric Mann-Whitney test for the quantitative variables and Fisher's exact test or *χ* ^2^ test for the qualitative variables. Factors associated with the outcome of hyperthyroidism were estimated using univariate analysis by logistic regression.

## 3. Result

### 3.1. Baseline Characteristics

The 51 patients were divided into two groups: GD with hepatic injury and GD with leukopenia. The baseline profiles are summarized in Table 1. No significant differences in age, course, and thyroid parameters were observed between the two groups. We also recorded the using of ATDs before lithium carbonate treatment (Table 1).

### 3.2. Effects of Treatment

**Figure 1:**
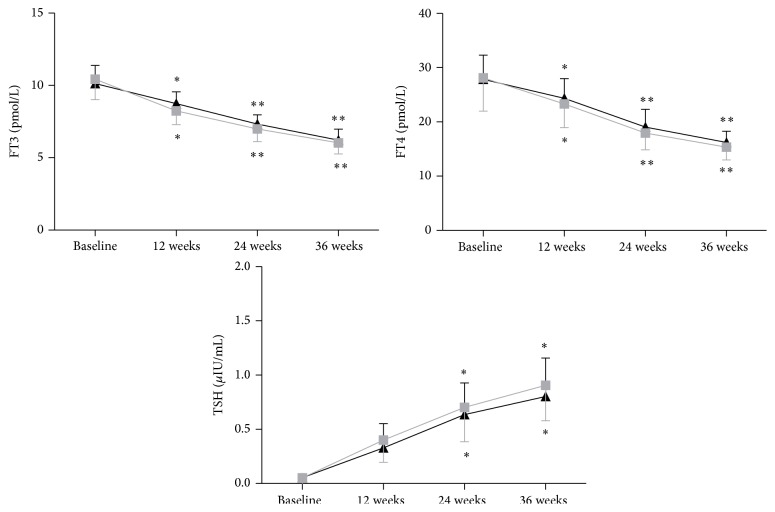
Outcome of serum thyroid hormone concentrations. Outcome of serums FT3, FT4, and TSH concentrations in GD with hepatic injury treated with lithium are shown by black line and that in GD with leukopenia treated with lithium is shown by gray line. Difference in the outcome of serums FT3, FT4, and TSH concentrations between baseline and lithium treatment versus baseline; ^*∗*^ *p* < 0.05; ^*∗∗*^ *p* < 0.01.

Fifty-one patients were treated with lithium carbonate 0.5–0.75 g/d. After 36 weeks of treatment, thyroid function was reevaluated. The FT3 and FT4 values in GD patients with hepatic injury before the lithium carbonate treatment were 10.12 ± 4.58 pmol/L and 27.46 ± 8.94 pmol/L and significantly decreased to 6.34 ± 1.42 pmol/L and 17.24 ± 4.31 pmol/L (*p* < 0.01) after the treatment for 36 weeks. In GD patients with leukopenia similar changes in FT3 and FT4 were observed, before 10.86 ± 5.35 pmol/L and 28.52 ± 10.23 pmol/L and after 6.15 ± 1.27 pmol/L and 16.31 ± 4.19 pmol/L (*p* < 0.01), respectively (Figure 1). The TSH value in GD patients with hepatic injury before the lithium carbonate treatment was 0.05 ± 0.05 mIU/L and significantly increased to 0.83 ± 0.52 mIU/L (*p* < 0.05) after the treatment for 36 weeks. In GD patients with leukopenia similar changes in TSH were observed, before 0.06 ± 0.07 mIU/L and after 0.92 ± 0.65 mIU/L (*p* < 0.05) (Figure 1).

Thirty-three GD patients with hepatic injury were treated with an additional hepatoprotective drug (Diammonium Glycyrrhizinate, Polyene Phosphatidylcholine). The levels of ALT, AST, and TBIL decreased and remained at the normal level after treatment for 36 weeks. Eighteen GD patients with leukopenia were treated with additional drugs to increase their white blood cells (Leucogen, Granulocyte colony-stimulating factor). The levels of WBC and N increased significantly and remained at the normal level after treatment for 36 weeks (data not shown).

According to patient's condition, it cannot be denied that we used additional glucocorticoids including prednisone and methylprednisolone for 37% of the patients in the short term and propranolol was prescribed in 94% of the patients (Table 2).

Blood pressure and blood glucose levels were not changed from the baseline and during the study period in the two groups (data not shown). One patient with mild heart failure (NYHA Functional Classification is Class II) improved, 1 patient sustained atrial fibrillation, and they received radioiodine therapy later.

### 3.3. Clinical Outcome

**Figure 2:**
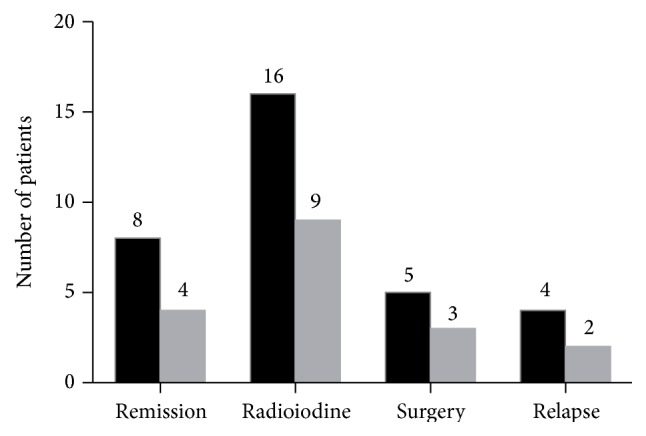
Clinical outcome. The number of patients with different outcomes in GD with hepatic injury (black) and in GD with leukopenia (gray).

After treatment for 36 weeks, the symptom of hyperthyroidism was controlled. The concentrations of thyroid hormones were decreased. Overall, 12 patients (23.5%) obtained clinical remission at the 1-year follow-up, 6 patients (11.8%) relapsed after withdrawal and continue to receive lithium carbonate treatment, 25 patients (49.0%) received radioiodine therapy after lithium carbonate treatment, and 8 patients (15.7%) received surgical treatment after lithium carbonate treatment (Figure 2). The liver function and white blood cells returned to normal levels in all patients.

### 3.4. Factors Associated with the Outcome of Hyperthyroidism

In comparing the remission with nonremission (radioiodine, surgery, and relapse) of the patients, we found that the failure to respond to lithium carbonate treatment may be correlated with several factors, including course of GD, thyroid hormone levels at baseline and 36 weeks, TRAb, and thyromegaly. No correlation was found between serum lithium concentration, lithium dose, age, treatment course, and glucocorticoid use (Table 3).

### 3.5. Serum Lithium Concentration and Side Effects

Serum lithium concentration in GD patients with hepatic injury before the lithium carbonate treatment was 0.45 ± 0.09 mmol/L and significantly increased to 0.56 ± 0.08 mmol/L (*p* < 0.05) after the treatment for 36 weeks. In GD patients with leukopenia similar changes in serum lithium level were observed, before 0.41 ± 0.06 mmol/L and after 0.54 ± 0.06 mmol/L (*p* < 0.05). We did not find that there was a change in serum concentrations of lithium after patients are treated with corticosteroids. No abnormalities of renal function and blood glucose were found, and the side effects of psoriasis have not been observed. Several patients appeared to have abdominal distention, vomiting, nausea, and so forth (Table 4). But the symptom disappeared soon after stopping lithium carbonate treatment. Afterwards, these patients received radioiodine therapy.

## 4. Discussion

Lithium carbonate is usually used to treat manic-depressive and depressive disorders [15], but lithium carbonate can also decrease the levels of thyroid hormones and lead to hypothyroidism during the treatment [16, 17]. So it is also used as second-line drug for the treatment of hyperthyroidism. The relevance of the relationship between lithium treatment and thyroid function [18, 19] is well documented.

Hepatic injury and leukopenia may occur in untreated patients with thyrotoxicosis and patients treated with MMT or PTU. GD with ATD-induced hepatic injury or leukopenia occurs frequently in clinical practice and treatment becomes more complex if these patients are unable to accept radioactive iodine therapy or surgery therapy; thus, we were prompted to analyze the clinical effect of lithium carbonate on these patients.

We enrolled 51 GD patients with hepatic injury or leukopenia in this study. The study showed that the symptoms of hyperthyroidism were controlled, thyroid hormones decreased to a certain extent in all patients, and thyroid function was maintained at normal levels in 12 patients (23.5%) who discontinued drug therapy after 36 weeks. Only 6 patients (11.8%) relapsed after lithium carbonate withdrawal for six months and continue to receive lithium carbonate treatment. Overall, 33 patients (64.7%) could not reach a satisfactory target; these patients were treated with radioactive iodine (25, 49.0%) or operation therapy (8, 15.7%). These findings indicate that lithium carbonate may be an effective therapeutic agent in the treatment of hyperthyroidism due to GD, especially in patients with hepatic injury or leukopenia. Additionally, our results indicated that lithium may have an effect on treating mild or moderate hyperthyroidism but a poor role in treating severe thyrotoxicosis.

In the present work, after 36 weeks of treatment with an additional hepatoprotective drug and a drug for increasing the white blood cells, liver function and white blood cell counts of patients improved. The levels of ALT, AST, and TBIL decreased in patients with hepatic injury, and the white blood cell counts increased in the patients with leukopenia. Lithium carbonate does not injure the liver in the routine dose and is a perfect substitution for ATD. Study has found that an additive effect of increasing white blood cells was observed in patients treated with lithium carbonate because it may modulate granulocytopoiesis [20]. Moreover, lithium salts can not only increase the level of CD34 but also induce granulocyte colony-stimulating factor and increase the number of neutrophils by stimulating bone marrow; thus, it is a good choice in patients with leucopenia or agranulocytosis [21–23].

On the other hand, failure of lithium carbonate treatment may be influenced by several factors, including course of GD, thyroid hormone levels at baseline and 36 weeks, TRAb, and large goiter. In this study, 25 patients required radioactive iodine treatment after being treated with lithium carbonate, and no patient presented with thyroid crisis. Lithium carbonate is used as an adjunct to radioiodine in the therapy of thyrotoxicosis. Data show that the thyroid iodine uptake rate (RAIU) can be significantly increased in patients treated with lithium carbonate [13]. Study showed the addition of lithium is beneficial for GD patients treated with RAI. Compared with the patients treated with RAI alone, the cure rate was higher in patients treated with RAI plus lithium, and lithium can reduce the serum FT4 level after treatment with radioiodine therapy [14]. Therefore, lithium helps make the RAI therapy more effective and the dose of ^131^I can be reduced [24].

Eight cases that required surgery after hyperthyroidism symptoms did not improve completely with lithium carbonate treatment, and these patients were stable during the intraoperative and postoperative periods. Akin et al. report that lithium carbonate is safe to treat patients with preoperative hyperthyroidism when ATDs are not effective and show adverse effects [25]. It is concluded that the using of lithium alone or in combination with ATDs is an effective way for controlling hyperthyroidism before operation [26, 27].

In general, lithium carbonate requires specific care as it has a narrow therapeutic range, with the therapeutic levels for psychiatric disorders ranging from 0.6 to 1.2 mmol/L. The serum monitoring of lithium levels is important for safety of patients and clinical effectiveness [28]. It is relatively safe that lithium carbonate was given 0.75 g/day. Several patients had some side effects in this study, such as abdominal distention, vomiting, nausea, and asthenia (Table 4); the symptoms disappeared when the lithium carbonate treatment was stopped. These patients were treated with radioiodine; other patients did not appear to have side effects. The kidney function of all patients was normal.

Inhibition of synthesis and secretion of thyroid hormones are the critical mechanism in the development of hypothyroidism [29–31]. Thyroid gland can take and concentrate lithium. It reduces thyroidal iodine uptake and also inhibits coupling of iodotyrosine, and it can promote the thyroxine conversion into triiodothyronine in thyroid gland [32]. In addition, the lithium salt can reduce thyroid adenylate cyclase activity, inhibit adenosine monophosphate, prevent the release of thyroid hormones, and decrease serum thyroid hormone levels [12]. However, studies show that lithium affects the hypothalamic-pituitary-thyroid axis to decrease thyroid hormone levels [29, 33].

In conclusion, lithium carbonate has multiple effects on the treatment of hyperthyroidism caused by GD. It is particularly suitable for patients with ATD-induced hepatic injury or leukopenia. Lithium carbonate could decrease the level of thyroid hormones. It is also effective for the preparation of radioactive iodine and surgical treatment in patients with thyrotoxicosis. Our clinical study shows that lithium carbonate is safe and has no severe side effect on the treatment of hyperthyroidism. Of course, the clinical research of lithium treatment on hyperthyroidism is rarely reported. Hence, we think that the antithyroid effect of lithium carbonate should be further studied, including pharmacologic mechanism,** c**ourse of treatment, long-term efficacy, and safety.

## 5. Conclusion

Lithium carbonate has effects on the treatment of mild-to-moderate hyperthyroidism caused by GD, and it is particularly suitable for patients with ATD-induced hepatic injury or leukopenia. It is also effective for the preparation of radioactive iodine and surgical treatment in patients with thyrotoxicosis.

## Figures and Tables

**Table 1:** Clinical and biochemical features of the study groups at baseline.

	GD with hepatic injury	GD with leukopenia	*p*
Patients (male/female), *n*	33 (5/28)	18 (3/15)	0.246
Age (month)	32.18 ± 15.21	35.25 ± 12.53	0.121
Duration (month)	12.34 ± 5.13	13.25 ± 6.51	0.435
Thyroid hormone			
FT3 (pmol/L)	10.12 ± 4.58	10.86 ± 5.35	0.421
FT4 (pmol/L)	27.46 ± 8.94	28.52 ± 10.23	0.224
TSH (mIU/L)	0.11 ± 0.05	0.12 ± 0.04	0.532
Antibody			
TRAb (U/L)	5.82 ± 7.11	6.13 ± 6.98	0.536
Positive TRAb (%)	30 (91)	17 (94)	0.452
Positive TPOAb (%)	11 (33)	7 (39)	0.251
Positive TGAb (%)	12 (36)	5 (28)	0.324
Liver function			
ALT (U/L)	183.95 ± 124.58	19.64 ± 12.73	0.000
AST (U/L)	135.32 ± 131.46	18.27 ± 10.44	0.000
TBIL (*μ*mol/L)	21.30 ± 11.69	12.38 ± 8.47	0.000
Blood cell count			
WBC (×10^9^/L)	6.49 ± 1.73	2.71 ± 0.58	0.000
N (×10^9^/L)	3.23 ± 0.89	1.35 ± 0.42	0.000
Thyromegaly (%)			
I	10 (30)	7 (39)	0.832
II	23 (70)	11 (61)	0.726
Used ATD (%)			
MMI	27 (81)	15 (83)	0.321
PTU	6 (19)	3 (17)	0.234

Data were expressed as mean ± standard deviation or percentage of the total. FT4, free thyroxine; FT3, free triiodothyronine; TSH, Thyroid Stimulating Hormone; TRAb, thyrotropin receptor antibody; TgAb, thyroglobulin antibodies; TPOAb, thyroglobulin antibodies; ALT, aminotransferase; AST, aspartate aminotransferase; WBC, white blood cell; N, neutrophil.

**Table 2:** Therapeutic measures for GD with hepatic injury and GD with leukopenia.

	GD with hepatic injury	GD with leukopenia
Patients (male/female), *n*	33 (5/28)	18 (3/15)
Lithium carbonate (%)		
50 mg/day	7 (21)	5 (28)
75 mg/day	26 (79)	13 (72)
Hepatoprotective (%)		
Diammonium Glycyrrhizinate	22 (67)	0
Polyene Phosphatidylcholine	16 (48)	0
Leucocyte drug (%)		
Leucogen	0	16 (89)
GCSF	0	3 (17)
Glucocorticoids (%)		
Prednisone	9 (27)	5 (28)
Methylprednisolone	3 (9)	2 (11)
Propranolol (%)	32 (97)	16 (89)

Data were expressed as percentage of the total.

**Table 3:** Factors associated with the outcome of hyperthyroidism.

	OR	95% CI	*p*
Age	0.867	0.924–1.382	0.651
Course of GD	4.080	1.108–15.020	0.034
Gender (male/female)	0.189	0.014–1.473	0.091
Serum FT3 at baseline	0.049	0.006–0.431	0.007
Serum FT4 at baseline	0.031	0.027–0.642	0.015
Serum TSH at baseline	0.246	0.023–2.163	0.249
Serum FT3 at 36 weeks	0.039	0.003–0.453	0.010
Serum FT4 at 36 weeks	0.055	0.004–0.960	0.047
Serum TSH at 36 weeks	0.099	0.008–1.288	0.077
Positive TRAb	0.032	0.003–0.433	0.009
Positive TPOAb	0.139	0.012–1.684	0.121
Positive TGAb	0.179	0.015–2.140	0.174
Lithium dose	0.212	0.018–2.467	0.215
Serum lithium concentration	0.324	0.036–1.539	0.387
Propranolol	0.287	0.025–1.283	0.264
Thyromegaly	0.022	0.016–0.352	0.013

FT4, free thyroxine; FT3, free triiodothyronine; TSH, thyroid stimulating hormone; TRAb, thyrotropin receptor antibody; TgAb, thyroglobulin antibodies; TPOAb, thyroglobulin antibodies.

**Table 4:** Adverse reaction of lithium treatment.

	GD with hepatic injury	GD with leukopenia
Number of patients	33	18
Neurological symptoms (%)		
Mental confusion	0	0
Seizures	0	0
Drowsiness	0	0
Dizziness	0	0
Tremor	0	0
Gastrointestinal symptoms (%)		
Nausea	3 (9.0)	2 (11.1)
Vomiting	1 (3.0)	0
Diarrhea	0	1 (5.5)
Abdominal distention	1 (3.0)	0
Constipation	0	0
Other symptoms (%)		
Asthenia	1 (3.0)	0
Malaise	0	1 (5.5)
Blurred vision	0	0

Data were expressed as percentage of the total.

## References

[1] Brent G. A. (2008). Clinical practice. Graves' disease. *The New England Journal of Medicine*.

[2] Williams K. V., Nayak S., Becker D., Reyes J., Burmeister L. A. (1997). Fifty years of experience with propylthiouracil-associated hepatotoxicity: what have we learned?. *The Journal of Clinical Endocrinology and Metabolism*.

[3] Liaw Y.-F., Huang M.-J., Fan K.-D., Li K.-L., Wu S.-S., Chen T.-J. (1993). Hepatic injury during propylthiouracil therapy in patients with hyperthyroidism. A cohort study. *Annals of Internal Medicine*.

[4] Woeber K. A. (2002). Methimazole-induced hepatotoxicity. *Endocrine Practice*.

[5] Cooper D. S. (2005). Antithyroid drugs. *The New England Journal of Medicine*.

[6] Takata K., Kubota S., Fukata S. (2009). Methimazole-induced agranulocytosis in patients with Graves' disease is more frequent with an initial dose of 30 mg daily than with 15 mg daily. *Thyroid*.

[7] Watanabe N., Narimatsu H., Noh J. Y. (2012). Antithyroid drug-induced hematopoietic damage: a retrospective cohort study of agranulocytosis and pancytopenia involving 50,385 patients with Graves' disease. *The Journal of Clinical Endocrinology and Metabolism*.

[8] Kharlip J., Cooper D. S. (2009). Recent developments in hyperthyroidism. *The Lancet*.

[9] Tajiri J., Noguchi S. (2004). Antithyroid drug-induced agranulocytosis: special reference to normal white blood cell count agranulocytosis. *Thyroid*.

[10] Bahn R. S., Burch H. B., Cooper D. S. (2011). Hyperthyroidism and other causes of thyrotoxicosis: management guidelines of the American Thyroid Association and American Association of Clinical Endocrinologists. *Thyroid*.

[11] Kristensen O., Andersen H. H., Pallisgaard G. (1976). Lithium carbonate in the treatment of thyrotoxicosis. A controlled trial. *The Lancet*.

[12] Lazarus J. H. (2009). Lithium and thyroid. *Best Practice and Research: Clinical Endocrinology and Metabolism*.

[13] Bogazzi F., Bartalena L., Brogioni S. (1999). Comparison of radioiodine with radioiodine plus lithium in the treatment of Graves' hyperthyroidism. *The Journal of Clinical Endocrinology and Metabolism*.

[14] Bogazzi F., Giovannetti C., Fessehatsion R. (2010). Impact of lithium on efficacy of radioactive iodine therapy for Graves' disease: a cohort study on cure rate, time to cure, and frequency of increased serum thyroxine after antithyroid drug withdrawal. *The Journal of Clinical Endocrinology and Metabolism*.

[15] Bowden C. L. (1998). Key treatment studies of lithium in manic-depressive illness: efficacy and side effects. *The Journal of Clinical Psychiatry*.

[16] Parker P. E., Walter-Ryan W. G., Pittman C. S., Folks D. G. (1986). Lithium treatment of hyperthyroidism and mania. *The Journal of Clinical Psychiatry*.

[17] Emerson C. H., Dysno W. L., Utiger R. D. (1973). Serum thyrotropin and thyroxine concentrations in patients recieving lithium carbonate. *The Journal of Clinical Endocrinology and Metabolism*.

[18] Jefferson J. W. (1979). Lithium carbonate-induced hypothyroidism. Its many faces. *Journal of the American Medical Association*.

[19] Bocchetta A., Loviselli A. (2006). Lithium treatment and thyroid abnormalities. *Clinical Practice and Epidemiology in Mental Health*.

[20] Gallicchio V. S., Chen M. G. (1980). Modulation of murine pluripotential stem cell proliferation in vivo by lithium carbonate. *Blood*.

[21] Lyman G. H., Williams C. C., Preston D. (1980). The use of lithium carbonate to reduce infection and leukopenia during systemic chemotherapy. *The New England Journal of Medicine*.

[22] Petrini M., Azzarà A. (2012). Lithium in the treatment of neutropenia. *Current Opinion in Hematology*.

[23] Meisler A. I. (1977). Lithium carbonate in chemotherapy-induced neutropenia. *The New England Journal of Medicine*.

[24] Bal C. S., Kumar A., Pandey R. M. (2002). A randomized controlled trial to evaluate the adjuvant effect of lithium on radioiodine treatment of hyperthyroidism. *Thyroid*.

[25] Akin F., Yaylali G. F., Bastemir M. (2008). The use of lithium carbonate in the preparation for definitive therapy in hyperthyroid patients. *Medical Principles and Practice*.

[26] Takami H. (1994). Lithium in the preoperative preparation of Graves' disease. *International Surgery*.

[27] Mochinaga N., Eto T., Maekawa Y., Tsunoda T., Kanematsu T., Izumi M. (1994). Successful preoperative preparation for thyroidectomy in Graves' disease using lithium alone: report of two cases. *Surgery Today*.

[28] Wijeratne C., Draper B. (2011). Reformulation of current recommendations for target serum lithium concentration according to clinical indication, age and physical comorbidity. *The Australian and New Zealand Journal of Psychiatry*.

[29] Lazarus J. H. (1998). The effects of lithium therapy on thyroid and thyrotropin-releasing hormone. *Thyroid*.

[30] Spaulding S. W., Burrow G. N., Bermudez F., Himmelhoch J. M. (1972). The inhibitory effect of lithium on thyroid hormone release in both euthyroid and thyrotoxic patients. *The Journal of Clinical Endocrinology and Metabolism*.

[31] Lazarus J. H., Addison G. M., Richards A. R., Owen G. M. (1974). Treatment of thyrotoxicosis with lithium carbonate. *The Lancet*.

[32] Frankenfeld T. G. P., Corrêa da Costa V. M., Nascimento-Saba C. C. A. (2002). Thyroid and pituitary thyroxine-5′-deiodinase activity and thyrotrophin secretion in lithium-treated rats. *The Journal of Endocrinology*.

[33] Lombardi G., Panza N., Biondi B. (1993). Effects of lithium treatment on hypothalamic-pituitary-thyroid axis: a longitudinal study. *Journal of Endocrinological Investigation*.

